# Dataset of 111 metagenome-assembled genomes from cattle manure, soil and manured soil samples

**DOI:** 10.1016/j.dib.2025.111748

**Published:** 2025-06-03

**Authors:** Eduardo Pérez-Valera, Dana Elhottová

**Affiliations:** a INRAE, University Bourgogne, Institut Agro Dijon, Department of Agroecology, 17 rue de Sully, Dijon, 21000, France; b Institute of Soil Biology and Biogeochemistry, Biology Centre of the Czech Academy of Sciences, Na Sádkách 7, České Budějovice, 370 05, Czech Republic

**Keywords:** *Acinetobacter*, *Pseudomonas*, CHROMagar acinetobacter, Non-fermenting Gram-negative bacteria, Opportunistic pathogens

## Abstract

This data report presents 111 metagenome-assembled genomes (MAGs) reconstructed from manure, soil and manured soil samples from microcosms after enriching for non-fermenting Gram-negative bacteria (NFGNB). Two independent microcosm experiments were conducted to investigate the spread of NFGNB from the fresh manure of dairy cows under antibiotic prophylaxis to the pasture soil of two organic farms. After sampling the microcosms on days 2, 14 and 28, the manure and soil samples were plated in duplicate on CHROMagar Acinetobacter medium for NFGNB enrichment and incubated at 28°C for 24 h. DNA was extracted from the cultures and sequenced using the Illumina NovaSeq 6000 platform with 150-bp paired-end reads. Reads were assembled with metaSPAdes both individually and by co-assembly. MAGs were reconstructed using MetaBAT, MaxBin, SemiBin2, COMEbin, and AVAMB, and then de-replicated at >95 % ANI (pairwise comparisons) using dRep. A total of 111 MAGs of at least medium quality (MIMAG standard) were obtained. These included 10 high-quality MAGs (>90 % completeness, <5 % contamination, rRNA genes and tRNA for at least 18 amino acids), 47 putative high-quality MAGs (>90 % completeness, <5 % contamination) and 54 medium-quality MAGs (>50 % completeness, <10 % contamination). The FASTA files of the MAGs as well as their taxonomic identifications, completeness and contamination, origin, genomic statistics and rRNA sequences are publicly available in a Zenodo dataset and the genomes in the NCBI database. The majority of MAGs (99) were assigned to Pseudomonadota, mainly *Pseudomonas* (28 MAGs), *Stenotrophomonas* (20 MAGs) and *Acinetobacter* (18 MAGs), while the remaining 12 MAGs belonged to Bacteroidota. Most MAGs (44) were of manure origin, followed by manured soil (38 MAGs) and soil (29 MAGs). High-quality MAGs were predominantly obtained from manure (6 high-quality, 21 putative high-quality), compared to manured soil (3 high-quality, 12 putative high-quality) and soil (1 high-quality, 14 putative high-quality). By providing their MAGs, this dataset offers a valuable resource for researchers investigating the genomic characteristics associated with the survival, environmental dispersal and ecological role of potentially hazardous NFGNB species in soil, particularly following the application of antibiotic-treated animal manure, and for comparative genomics studies in related environments.

Specifications Table

**Table:** 

Subject	Biology
Specific subject area	Metagenome-assembled genomes of non-fermenting Gram-negative bacteria from manure, soil and manured soil samples
Type of data	Table, Figure and FASTA files of MAGs
Data collection	Genomic DNA was extracted from bacteria from microcosms combining soil and dairy cow manure, following enrichment for NFGNB on CHROMagar Acinetobacter. Genomic DNA was isolated using the Fast DNA Spin Kit and sequenced on an Illumina Novaseq 6000 platform. Sequence reads were quality-checked and assembled using metaSPAdes. MAGs representing 111 non-redundant bacterial species were reconstructed using MetaBAT, MaxBin, SemiBin2, COMEbin, and AVAMB, and de-replicated at >95 % ANI (pairwise comparisons) using dREP
Data source location	Location: České Budějovice, Czech Republic. Soil and manure samples for the microcosm experiment were located at 48 °North, 14 °East
Data accessibility	Repository name: Dataset of 111 metagenome-assembled genomes from cattle manure, soil and manured soil samples Data identification number: NCBI BioProject PRJNA1231077, ZENODO 10.5281/zenodo.15309541 Direct URL to data: NCBI: https://www.ncbi.nlm.nih.gov/bioproject/PRJNA1231077 ZENODO: 10.5281/zenodo.15309541
Related research article	[1] P. Sardar, D. Elhottová, E. Pérez-Valera, Soil-specific responses in the antibiotic resistome of culturable Acinetobacter spp. and other non-fermentative Gram-negative bacteria following experimental manure application. FEMS Microbiol. Ecol. 99 (2023) fiad148. https://doi.org/10.1093/femsec/fiad148. The metagenomic data and a description of the microcosm set up can be found in [1].

## 1 Value of the Data

• The dataset provides a comprehensive collection of 111 non-redundant MAGs from non-fermenting Gram-negative bacteria (NFGNB) isolated from soil and manure samples.
• This collection comprises 10 high-quality (MIMAG standard), 47 putative high-quality, and 54 medium-quality MAGs, representing 17 different genera that include *Pseudomonas* (28 MAGs), *Stenotrophomonas* (20 MAGs) and *Acinetobacter* (18 MAGs).
• A total of 44 MAGs originated from manure, 38 from manured soil and 29 from soil. High-quality MAGs were predominantly obtained from manure (6 high-quality, 21 putative high-quality), compared to manured soil (3 high-quality, 12 putative high-quality) and soil (1 high-quality, 14 putative high-quality).
• The genomic resources provided in this dataset can serve as a basis for future research on the survival, dissemination in the environment, and ecological role of bacterial species of potential clinical relevance.

## 2 Background

Non-fermenting Gram-negative bacteria (NFGNB) are becoming a growing concern due to their role in antimicrobial resistance and as healthcare-associated pathogens [2]. *Acinetobacter* spp. and other NFGNB such as *Pseudomonas* are widely distributed in nature, particularly in soil, water and the gastrointestinal tract of animals. These bacteria exhibit inherent resistance to many antibiotics [3] and readily acquire additional resistance mechanisms [4]. This adaptability has made them a key focus in clinical settings [5]. Previous research suggests that fresh manure from antibiotic-treated cattle enriches the soil with antibiotic resistance genes [6]. *Acinetobacter* spp. is known to thrive in soil following manure application, being a main actor that potentially contributes to the spread of antibiotic resistance in the environment [7]. In our previous study [1], we performed shotgun metagenomic sequencing to analyse the abundance, taxonomic identification and composition of the antibiotic resistome of NFGNB in manure, soil and manured soil samples. Here, we reconstructed 111 non-redundant MAGs from the metagenomes that account for approximately 91 % of the sequencing reads on average. The MAGs we provide can help to unravel the ecological and genomic mechanisms responsible for their spread and the spread of antibiotic resistance in the environment.

## 3 Data Description

**Fig. 1:**
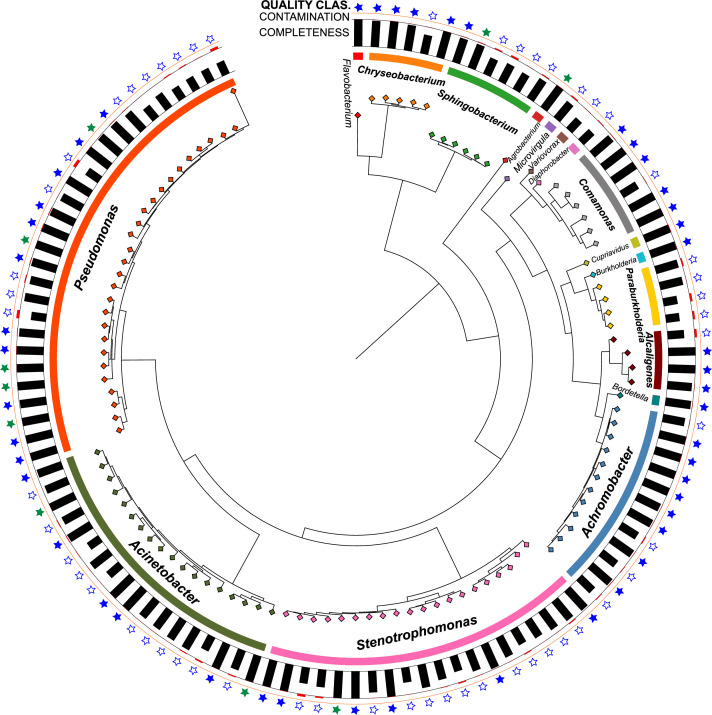
Phylogenomic tree and quality assessment of 111 metagenome-assembled genomes (MAGs) reconstructed from manure, soil and manured soil samples after enrichment with CHROMagar Acinetobacter. The tree was constructed using fastree on the MSA alignment by GTDB-tk using 120 concatenated single-copy bacterial genes. Outer rings show MAG quality classification: filled green stars indicate high-quality MAGs (MIMAG standard), filled blue stars indicate putative high-quality MAGs (>90 % completeness and <5 % contamination), while unfilled stars represent medium-quality MAGs (>50 % completeness and <10 % contamination). Red bars beneath the stars indicate contamination levels (0–10 %), whereas black bars represent completeness (50–100 %) as indicated by CheckM2.

The dataset contains 111 non-redundant (ANI >95 %) metagenome-assembled genomes (MAGs), all of which meet at least the MIMAG standard for medium quality (>50 % completeness and <10 % contamination) defined by Bowers et al. [8]. From these, we report 10 MAGs of high quality (>90 % completeness, <5 % contamination, encoding all 5S, 16S and 23S rRNAs genes and tRNAs for at least 18 of the 20 amino acids), 47 MAGs of putative high quality (>90 % completeness and <5 % contamination) and 54 MAGs of medium quality. High-quality MAGs were almost complete (98.4 ± 0.6 %, average ± SD) and showed low contamination (0.5 ± 0.6 %). Putative high-quality MAGs had a completeness of 97.5 ± 3 % and contamination of 0.6 ± 0.6 %. The remaining medium-quality MAGs had an average completeness of 70 ± 13 % and contamination of 2.6 ± 2.7 %. The dataset comprises MAGs assembled DNA reads as compressed FASTA files (.fasta.gz) and associated metadata in an Excel spreadsheet (MAGs_data.xlsx). The MAGs have been deposited in NCBI under the BioProject PRJNA1231077 and in Zenodo under https://doi.org/10.5281/zenodo.15309541. The Excel file “MAGs_data.xlsx”, included in the Zenodo dataset, details the following information: MAGs name, origin (manure, soil or manured soil), experiment (whether soil S or B), sample name, binning method, detection of 5S, 16S and 23S rRNA genes, number of nucleotides in the tRNAs, quality metrics (completeness, contamination, GC content, N50, genome size, scaffold and contig count, N90, L50 and L90), taxonomic affiliations predicted with GTDB-Tk, including best matching taxonomy and % ANI for the closest placement in GTDB (for MAGs with ANI >95 %), mapping reads in % average and maximum in a sample, NCBI information (i.e., SRA and BioSample accessions, coverage), and 16S rRNA-based identification and sequence. The tools used to extract each feature from the MAGs are also included in the Excel file. A summary of main MAG characteristics is given in Table 1. A phylogenomic tree (Fig. 1) illustrates the relationships among MAGs, their genome completeness, the percentage of contamination, and whether each MAG is a high-, putative high- or medium-quality MAG, as described above.

**Table 1:** General characteristics of the reconstructed NFGNB MAGs from manure, soil or manured soil (M. soil) samples. Taxonomic classifications at the phylum and genus levels were performed using GTDB-Tk [17], and ANI values to the closest reference genomes are provided. ANI values from GTDB-Tk are only reported for MAGs identified at the species level (i.e., all ANIs provided are > 95 %). MAG quality was assessed following the MIMAG standard [8], modified to include putative high-quality MAGs as those with completeness >90 % and contamination <5 %. MAGs names were assigned based on the binning method. MAGs meeting the high-quality MIMAG standard are indicated in bold. A more detailed table, including NCBI accessions and full genome information, is available in the Zenodo dataset (https://doi.org/10.5281/zenodo.15309541).

MAG	Origin	Original sample		Phylum	Genus (GTDB)	Species (GTDB)	Closest genome ANI (%)	MAG Quality	Completeness (%)	Contamination (%)	Binning Method	Genome size (bp)
**S21**	Manure	GT28SEX		Pseudomonadota	*Achromobacter*	Unknown	Not assigned	pHigh	100	1.21	SemiBin2	6,542,997
**S2**	M. soil	GT14BC2		Pseudomonadota	*Achromobacter*	Unknown	Not assigned	pHigh	99.9	0.38	SemiBin2	5,722,719
**S5**	Manure	GT14BEX		Pseudomonadota	*Achromobacter*	Unknown	Not assigned	pHigh	94.5	1.26	SemiBin2	6,165,035
**V20**	Manure	GT28SEXb		Pseudomonadota	*Achromobacter*	Unknown	Not assigned	pHigh	93.2	0.76	AVAMB	5,921,085
**C7**	M. soil	GT28BC2		Pseudomonadota	*Achromobacter*	Unknown	Not assigned	Medium	72.2	3.29	COMEbin	5,283,433
**V10**	Manure	GT28BEXa		Pseudomonadota	*Achromobacter*	*A. denitrificans*	99.18	pHigh	100	0.45	AVAMB	6,582,530
**V14**	Soil	GT28SAa		Pseudomonadota	*Achromobacter*	*A. kerstersii*	98.84	pHigh	93.2	0.71	AVAMB	5,861,663
**V18**	Soil	GT28Sac		Pseudomonadota	*Achromobacter*	*A. marplatensis*	97.67	pHigh	94.5	0.84	AVAMB	6,267,628
**M5**	Manure	GT28BEXc		Pseudomonadota	*Achromobacter*	*A. mucicolens*	98.9	pHigh	99.7	0.3	MaxBin	5,857,610
**C5**	Manure	GT14SEX		Pseudomonadota	*Achromobacter*	*A. piechaudii*	98.26	Medium	68.5	1.58	COMEbin	4,666,491
**C20**	M. soil	GT14BC2		Pseudomonadota	*Achromobacter*	*A. spanius*	95.09	Medium	67.6	2.39	COMEbin	4,643,289
**V41**	Manure	GT14BEXa		Pseudomonadota	*Achromobacter*	*A. veterisilvae*	97.99	Medium	81.7	1.03	AVAMB	5,818,176
**V37**	Manure	GT2BEXb		Pseudomonadota	*Acinetobacter*	Unknown	Not assigned	Medium	88.5	0.25	AVAMB	2,854,542
**V23**	M. soil	GT2BC2b		Pseudomonadota	*Acinetobacter*	Unknown	Not assigned	Medium	82.5	0.77	AVAMB	2,829,191
**C6**	Manure	GT14SEX		Pseudomonadota	*Acinetobacter*	Unknown	Not assigned	Medium	50.7	1.24	COMEbin	1,794,093
**V21**	M. soil	GT2BC2b		Pseudomonadota	*Acinetobacter*	*A. amyesii*	97.47	pHigh	94.6	0.39	AVAMB	3,279,076
**C16**	Manure	GT2SEX		Pseudomonadota	*Acinetobacter*	*A. baumannii*	97.68	Medium	75.8	3.3	COMEbin	3,202,536
**C15**	Manure	GT2BEX		Pseudomonadota	*Acinetobacter*	*A. bohemicus*	96.02	Medium	81.2	0.49	COMEbin	2,634,877
**M13**	Soil	GT2Sab		Pseudomonadota	*Acinetobacter*	*A. calcoaceticus*	96.32	pHigh	99.9	0.13	MetaBAT	3,801,409
**C10**	M. soil	GT28BC2		Pseudomonadota	*Acinetobacter*	*A. calcoaceticus*	97.12	Medium	56.7	4.79	COMEbin	2,263,300
**V36**	Manure	GT2BEXa		Pseudomonadota	*Acinetobacter*	*A. faecalis*	98.95	pHigh	95.2	1.75	AVAMB	2,344,785
**S3**	**M. soil**	**GT14BC2**		**Pseudomonadota**	***Acinetobacter***	***A. gandensis***	**98.68**	**High**	**100**	**0.36**	**SemiBin2**	**3,194,030**
**S24**	M. soil	GT2BC2		Pseudomonadota	*Acinetobacter*	*A. guillouiae*	97.73	Medium	86.7	2.58	SemiBin2	3,781,671
**S32**	Manure	GT2SEX		Pseudomonadota	*Acinetobacter*	*A. johnsonii*	95.78	pHigh	99.9	0.41	SemiBin2	3,375,171
**V35**	Manure	GT2SEXc		Pseudomonadota	*Acinetobacter*	*A. pseudolwoffii*	97.93	Medium	84.0	0.88	AVAMB	2,458,038
**V8**	Soil	GT28Baa		Pseudomonadota	*Acinetobacter*	*A. schindleri*	97.71	pHigh	100	0.16	AVAMB	3,060,448
**S29**	M. soil	GT2SC2		Pseudomonadota	*Acinetobacter*	Acinetobacter sp002135435	98.93	Medium	81.2	2.28	SemiBin2	3,040,298
**V38**	Manure	GT2BEXc		Pseudomonadota	*Acinetobacter*	Acinetobacter sp002365595	98.16	Medium	85.6	0.37	AVAMB	2,686,145
**S30**	Manure	GT2SEX		Pseudomonadota	*Acinetobacter*	Acinetobacter sp013417555	95.76	Medium	58.4	0.37	SemiBin2	1,796,683
**M8**	**Manure**	**GT2SEXc**		**Pseudomonadota**	***Acinetobacter***	***A. vivianii***	**97.38**	**High**	**100**	**0.12**	**MaxBin**	**3,884,090**
**C18**	**Soil**	**GT14BA**		**Pseudomonadota**	***Agrobacterium***	***A. fabacearum***	**98.72**	**High**	**99.9**	**1.01**	**COMEbin**	**5,070,611**
**M10**	Manure	GT2SEXc		Pseudomonadota	*Alcaligenes*	Unknown	Not assigned	Medium	61.1	7.74	MaxBin	3,792,446
**M14**	Manure	GT2SEXa		Pseudomonadota	*Alcaligenes*	*Alcaligenes faecalis*	98.45	pHigh	100	0.57	MetaBAT	4,114,606
**V12**	Manure	GT28BEXc		Pseudomonadota	*Alcaligenes*	*Alcaligenes nematophilus*	97.5	pHigh	90.2	0.6	AVAMB	3,979,026
**V5**	Manure	GT14SEXb		Pseudomonadota	*Alcaligenes*	Alcaligenes sp023425645	97.38	pHigh	99.3	0.96	AVAMB	3,774,399
**C14**	Manure	GT28SEX		Pseudomonadota	*Bordetella*	*Bordetella trematum*	99.59	pHigh	93.7	0.7	COMEbin	4,161,353
**V17**	Soil	GT28Sac		Pseudomonadota	*Burkholderia*	*Burkholderia contaminans*	98.43	pHigh	97.8	0.49	AVAMB	8,062,764
**V16**	Soil	GT28Sac		Bacteroidota	*Chryseobacterium*	*C. culicis*	95.33	Medium	70.3	0.4	AVAMB	4,177,414
**V24**	M. soil	GT2BC2c		Bacteroidota	*Chryseobacterium*	*C. jejuense*	95.24	pHigh	100	0.61	AVAMB	5,212,491
**S22**	M. soil	GT2BC2		Bacteroidota	*Chryseobacterium*	*C. joostei*	96.37	pHigh	94.8	0.13	SemiBin2	4,459,574
**V31**	Soil	GT2Sac		Bacteroidota	*Chryseobacterium*	*C. rhizosphaerae*	98.08	pHigh	95.9	0.1	AVAMB	5,097,737
**V15**	Soil	GT28SAa		Bacteroidota	*Chryseobacterium*	Chryseobacterium sp900156935	99.37	pHigh	99.9	0.14	AVAMB	5,184,267
**S15**	M. soil	GT28BC2		Pseudomonadota	*Comamonas*	Unknown	Not assigned	pHigh	90.7	1.07	SemiBin2	4,586,957
**C12**	Manure	GT28BEX		Pseudomonadota	*Comamonas*	Unknown	Not assigned	Medium	65.9	1.09	COMEbin	1,814,831
**V2**	Soil	GT14Sac		Pseudomonadota	*Comamonas*	*C. koreensis*	98.91	pHigh	100	0.08	AVAMB	4,875,248
**M15**	Manure	GT14BEXb		Pseudomonadota	*Comamonas*	*C. sp002472915*	98.21	pHigh	100	0.27	MetaBAT	4,874,396
**S28**	M. soil	GT2SC2		Pseudomonadota	*Comamonas*	*C. testosteroni*	98.83	pHigh	100	0.2	SemiBin2	5,095,908
**M11**	Manure	GT14BEXc		Pseudomonadota	*Comamonas*	*C. tsuruhatensis*	98.17	pHigh	100	0	MaxBin	6,154,020
**S14**	M. soil	GT28BC2		Pseudomonadota	*Cupriavidus*	Cupriavidus sp000955785	96.49	pHigh	99.8	1.22	SemiBin2	7,429,278
**C8**	M. soil	GT28BC2		Pseudomonadota	*Diaphorobacter*	*D. nitroreducens*	98.31	Medium	54.3	1.95	COMEbin	2,384,275
**V26**	Soil	GT2Sab		Bacteroidota	*Flavobacterium*	Flavobacterium sp002303885	98.08	pHigh	99.7	0.08	AVAMB	5,375,231
**C9**	M. soil	GT28BC2		Pseudomonadota	*Microvirgula*	Unknown	Not assigned	Medium	77.4	4.05	COMEbin	2,880,378
**C19**	Soil	GT14BA		Pseudomonadota	*Paraburkholderia*	*P. hospita*	98.85	Medium	84.9	4.33	COMEbin	6,736,010
**C17**	Soil	GT14BA		Pseudomonadota	*Paraburkholderia*	*P. nemoris*	97.71	Medium	52.2	6.96	COMEbin	2,366,553
**C2**	Soil	GT14BA		Pseudomonadota	*Paraburkholderia*	*P. nemoris*	98.59	Medium	50.4	6.75	COMEbin	4,222,632
**S1**	Soil	GT14BA		Pseudomonadota	*Paraburkholderia*	*P. terricola*	99.26	Medium	85.3	0.25	SemiBin2	5,799,680
**V33**	Manure	GT2SEXa		Pseudomonadota	*Pseudomonas*	Unknown	Not assigned	pHigh	97.1	0.07	AVAMB	5,154,236
**S27**	Soil	GT2SA		Pseudomonadota	*Pseudomonas*	Unknown	Not assigned	Medium	83.7	0.97	SemiBin2	4,956,356
**M2**	M. soil	GT28BC2c		Pseudomonadota	*Pseudomonas*	Unknown	Not assigned	Medium	55.6	9.21	MaxBin	5,220,937
**M7**	M. soil	GT2SC2b		Pseudomonadota	*Pseudomonas*	Unknown	Not assigned	Medium	52.1	8.53	MaxBin	2,442,360
**C4**	M. soil	GT14SC2		Pseudomonadota	*Pseudomonas*	*P. alloputida*	96.52	Medium	52.5	2.46	COMEbin	3,936,810
**S31**	**Manure**	**GT2SEX**		**Pseudomonadota**	***Pseudomonas***	***P. capeferrum***	**99.59**	**High**	**99.5**	**0.51**	**SemiBin2**	**5,724,253**
**S26**	Manure	GT2SEX		Pseudomonadota	*Pseudomonas*	*P. helleri*	97.48	pHigh	99.7	0.14	SemiBin2	5,310,282
**S18**	**Manure**	**GT28BEX**		**Pseudomonadota**	***Pseudomonas***	***P. kermanshahensis***	**96.83**	**High**	**92.3**	**1.06**	**SemiBin2**	**5,678,766**
**V28**	Soil	GT2Sac		Pseudomonadota	*Pseudomonas*	*P. laurylsulfatiphila*	99.66	pHigh	98.4	0.07	AVAMB	6,282,357
**M9**	Manure	GT2SEXc		Pseudomonadota	*Pseudomonas*	*P. oleovorans*	96.86	pHigh	100	0.2	MaxBin	5,542,180
**S9**	**Manure**	**GT14SEX**		**Pseudomonadota**	***Pseudomonas***	***P. palmensis***	**98.79**	**High**	**100**	**0.16**	**SemiBin2**	**5,571,667**
**V32**	M. soil	GT2SC2c		Pseudomonadota	*Pseudomonas*	*P. protegens*	98.93	Medium	82.9	3.03	AVAMB	5,989,150
**V29**	Soil	GT2Sac		Pseudomonadota	*Pseudomonas*	*P. protegens*	96.47	pHigh	93.0	1.19	AVAMB	6,564,374
**V25**	Soil	GT2SAa		Pseudomonadota	*Pseudomonas*	*P. putida*	97.21	Medium	55.8	0.18	AVAMB	3,376,573
**C1**	Soil	GT14SA		Pseudomonadota	*Pseudomonas*	*P. putida*	97.76	Medium	55.5	9.77	COMEbin	1,188,256
**V22**	M. soil	GT2BC2b		Pseudomonadota	*Pseudomonas*	*P. putida*	99.25	pHigh	95.4	0.41	AVAMB	5,609,907
**V27**	Soil	GT2Sac		Pseudomonadota	*Pseudomonas*	*P. putida*	98	Medium	64.2	0.09	AVAMB	4,083,299
**C3**	M. soil	GT14SC2		Pseudomonadota	*Pseudomonas*	*P. putida*	97.94	Medium	50.3	2.47	COMEbin	3,664,077
**S4**	M. soil	GT14BC2		Pseudomonadota	*Pseudomonas*	*P. shirazensis*	97.29	Medium	85.3	0.38	SemiBin2	4,717,260
**S19**	M. soil	GT28SC2		Pseudomonadota	*Pseudomonas*	Pseudomonas sp000955815	99.69	pHigh	95.8	1.59	SemiBin2	5,181,704
**V19**	M. soil	GT28SC2b		Pseudomonadota	*Pseudomonas*	Pseudomonas sp001422615	96.85	Medium	51.8	0.63	AVAMB	2,968,572
**V9**	Soil	GT28Bab		Pseudomonadota	*Pseudomonas*	Pseudomonas sp001655615	97.24	pHigh	99.2	0.93	AVAMB	6,293,145
**S10**	Soil	GT28BA		Pseudomonadota	*Pseudomonas*	Pseudomonas sp020520285	99.26	Medium	89.7	0.22	SemiBin2	6,347,120
**M1**	**Manure**	**GT14SEXb**		**Pseudomonadota**	***Pseudomonas***	**Pseudomonas sp025837155**	**97.95**	**High**	**100**	**0**	**MaxBin**	**4,307,444**
**V39**	M. soil	GT14BC2b		Pseudomonadota	*Pseudomonas*	Pseudomonas sp029839275	96.98	pHigh	99.8	0.1	AVAMB	5,158,824
**S20**	M. soil	GT28SC2		Pseudomonadota	*Pseudomonas*	Pseudomonas sp900101695	98.83	pHigh	99.1	2.82	SemiBin2	5,501,807
**V11**	Manure	GT28BEXb		Pseudomonadota	*Pseudomonas*	Pseudomonas sp943914515	98.82	pHigh	94.1	1.56	AVAMB	6,141,918
**V42**	**Manure**	**GT14BEXb**		**Pseudomonadota**	***Pseudomonas***	***P. urmiensis***	**98.08**	**High**	**100**	**0.06**	**AVAMB**	**5,583,077**
**S16**	M. soil	GT28BC2		Bacteroidota	*Sphingobacterium*	Unknown	Not assigned	pHigh	93.3	0.87	SemiBin2	5,619,382
**S13**	M. soil	GT28BC2		Bacteroidota	*Sphingobacterium*	Unknown	Not assigned	Medium	70.0	0.41	SemiBin2	4,277,425
**S23**	**M. soil**	**GT2BC2**		**Bacteroidota**	***Sphingobacterium***	***S. paramultivorum***	**99.61**	**High**	**92.6**	**1.59**	**SemiBin2**	**5,603,380**
**C22**	M. soil	GT14BC2		Bacteroidota	*Sphingobacterium*	*S. siyangense*	97.46	Medium	65.4	3.9	COMEbin	4,334,548
**V3**	Soil	GT14Sac		Bacteroidota	*Sphingobacterium*	S. sp019969845	98.83	Medium	85.2	2.8	AVAMB	3,328,530
**C13**	Soil	GT28SA		Bacteroidota	*Sphingobacterium*	S. sp029542085	97.35	Medium	73.1	6.11	COMEbin	4,923,870
**S17**	Manure	GT28BEX		Pseudomonadota	*Stenotrophomonas*	Unknown	Not assigned	Medium	80.3	0.66	SemiBin2	3,719,936
**S6**	Manure	GT28BEX		Pseudomonadota	*Stenotrophomonas*	Unknown	Not assigned	Medium	73.1	0.24	SemiBin2	3,028,981
**M12**	Soil	GT28Bac		Pseudomonadota	*Stenotrophomonas*	Unknown	Not assigned	Medium	64.9	5.1	MetaBAT	3,278,791
**V6**	Manure	GT14SEXb		Pseudomonadota	*Stenotrophomonas*	*S. acidaminiphila*	98.57	pHigh	94.5	0.08	AVAMB	3,473,400
**C21**	M. soil	GT14BC2		Pseudomonadota	*Stenotrophomonas*	*S. bentonitica*	97.85	Medium	59.1	4.09	COMEbin	2,872,105
**V34**	Manure	GT2SEXb		Pseudomonadota	*Stenotrophomonas*	*S. geniculata*	98.2	pHigh	100	0	AVAMB	4,536,706
**M4**	Manure	GT28BEXb		Pseudomonadota	*Stenotrophomonas*	*S. hibiscicola*	98.03	pHigh	100	0.83	MaxBin	4,279,084
**V1**	Soil	GT14Sab		Pseudomonadota	*Stenotrophomonas*	*S. indicatrix*	97.05	pHigh	100	0.08	AVAMB	4,490,560
**M6**	M. soil	GT28SC2a		Pseudomonadota	*Stenotrophomonas*	*S. indicatrix*	95.08	Medium	59.6	8.16	MaxBin	2,601,569
**M3**	Manure	GT28BEXa		Pseudomonadota	*Stenotrophomonas*	*S. lactitubi*	95.19	pHigh	100	1.34	MaxBin	4,408,438
**V4**	Manure	GT14SEXa		Pseudomonadota	*Stenotrophomonas*	*S. maltophilia*	97.26	Medium	86.2	0.26	AVAMB	3,538,935
**S12**	M. soil	GT28BC2		Pseudomonadota	*Stenotrophomonas*	*S. maltophilia*	99.29	Medium	79.2	0.99	SemiBin2	3,729,623
**S25**	**M. soil**	**GT2BC2**		**Pseudomonadota**	***Stenotrophomonas***	***S. maltophilia***	**98.25**	**High**	**100**	**0**	**SemiBin2**	**4,563,766**
**V30**	Soil	GT2Sac		Pseudomonadota	*Stenotrophomonas*	*S. rhizophila*	96.26	pHigh	96.0	0.4	AVAMB	4,608,349
**V40**	M. soil	GT14BC2c		Pseudomonadota	*Stenotrophomonas*	*S. sepilia*	95.62	Medium	79.1	0.39	AVAMB	3,707,479
**V13**	Manure	GT28BEXc		Pseudomonadota	*Stenotrophomonas*	Stenotrophomonas sp002471015	97.88	Medium	63.7	3.24	AVAMB	2,761,634
**S8**	M. soil	GT14SC2		Pseudomonadota	*Stenotrophomonas*	Stenotrophomonas sp003086775	97.15	Medium	55.0	0.63	SemiBin2	3,218,735
**V7**	Manure	GT14SEXb		Pseudomonadota	*Stenotrophomonas*	Stenotrophomonas sp003484865	98.32	Medium	71.4	1.49	AVAMB	3,313,655
**C11**	Manure	GT28BEX		Pseudomonadota	*Stenotrophomonas*	Stenotrophomonas sp004348115	97.75	Medium	88.5	3.32	COMEbin	3,986,901
**S7**	M. soil	GT14SC2		Pseudomonadota	*Stenotrophomonas*	Stenotrophomonas sp030549615	96.88	Medium	80.7	0.54	SemiBin2	4,060,927
**S11**	Soil	GT28BA		Pseudomonadota	*Variovorax*	Variovorax sp000282635	99.08	Medium	52.6	0.56	SemiBin2	3,412,441

## 4 Experimental Design, Materials and Methods

Metagenome-assembled genomes (MAGs) were obtained from cattle manure, soil and manured soil samples after enrichment via cultivation in CHROMagar Acinetobacter (CHROMagar, Paris, France) as described in [1]. Briefly, microcosms combining fresh manure from a private dairy farm (under antibiotic prophylaxis) and soil from two organic farms were sampled after 2, 14 and 28 days of incubation. Five grams of each of the soil, manure or manured soil samples were used to inoculate plates in duplicate containing CHROMagar Acinetobacter. After incubating the plates at 28°C for 24h, microbial biomass was harvested by resuspending and centrifuging at 12,170 RCF for 5 min. Bacterial DNA was isolated using the Fast DNA Spin Kit (MP Biomedicals, Santa Ana, CA, USA) following the manufacturer's protocol. Shotgun metagenomic sequencing for a total of 52 samples was performed by Novogene (Hong Kong) on a NovaSeq 6000 instrument using 2×150 bp reads.

Raw reads were processed for quality-check, assembly and taxonomic analysis as described in [1]. Briefly, adapters, low-quality and contaminant reads were removed using BBMap and BBduk 38.96 [9]. For the construction of MAGs in this dataset, DNA reads were assembled individually (i.e., 52 assemblies), and co-assembled per soil, treatment and time (18 assemblies) using metaSPAdes 3.14.1. MAGs were obtained using multiple approaches that included AVAMB 4.1 [10], Semibin 2.1.0 [11], MetaBAT 2.17 [12], COMEbin 1.0.4 [13] and MaxBin 2.2.5 [14]. In the case of AVAMB, we used the individual assemblies following the recommendations. For the other binners, we used the co-assembled contigs. We assessed bin completeness and contamination using CheckM2 [15]. All MAGs were then clustered using dRep 3.5.0 [16] at > 95 % ANI (pairwise comparisons). One representative MAG from each cluster was chosen using the default score-based system in dRep. MAGs with >50 % completeness and <10 % contamination that met the MIMAG standard for at least medium quality were kept. MAGs were named sequentially according to the binning software used. Taxonomic assignment of MAGs was performed using GTDB-Tk 2.0 with the database GTDB R220 [17]. Species-level identification is only provided for MAGs with >95 % ANI to genomes in the GTDB reference database. A phylogenomic tree was constructed using fastree on the MSA alignments provided by GTDB-tk. The tree was visualized using iTol [18]. Complete (∼ 1,500 nucleotides) and near-complete (at least 1,200 nucleotides) 16S rRNA sequences were reconstructed from the raw fastq files using RiboTaxa 1.5 [19] using default parameters and linked to MAGs using MarkerMAG 1.1.28 [20].

## Limitations

The dataset includes 54 medium-quality MAGs (>50 % completeness, <10 % contamination), which may exhibit a higher degree of fragmentation compared to the 10 high-quality and 47 putative high-quality MAGs (>90 % completeness, <5 % contamination). These MAGs might limit certain types of genomic investigations, such as those requiring complete genomes or genes. Additionally, the use of a cultivation-based enrichment method targeting non-fermenting Gram-negative bacteria might have introduced a bias in the representation of the broader microbial community present in the original manure and soil samples. While this method was specifically chosen to focus on potentially risky NFGNB, other microbial groups might be underrepresented or absent from the resulting dataset.

## Ethics Statement

This research did not involve human subjects, animals, or any species requiring ethical approval.

## CRediT authorship contribution statement

**Eduardo Pérez-Valera:** Conceptualization, Methodology, Software, Validation, Formal analysis, Investigation, Resources, Data curation, Writing – original draft, Visualization, Supervision, Project administration, Funding acquisition. **Dana Elhottová:** Conceptualization, Investigation, Resources, Writing – review & editing, Project administration, Funding acquisition.

## Data Availability

ZenodoDataset of 111 metagenome-assembled genomes from cattle manure, soil and manured soil samples (Original data). NCBIDataset of 111 metagenome-assembled genomes from cattle manure and soil samples (Original data).
